# *IGF2*: Development, Genetic and Epigenetic Abnormalities

**DOI:** 10.3390/cells11121886

**Published:** 2022-06-10

**Authors:** Céline Sélénou, Frédéric Brioude, Eloïse Giabicani, Marie-Laure Sobrier, Irène Netchine

**Affiliations:** 1 Centre de Recherche Saint-Antoine, INSERM, Sorbonne Université, F-75012 Paris, France; celine.selenou@inserm.fr (C.S.); frederic.brioude@aphp.fr (F.B.); eloise.giabicani@aphp.fr (E.G.); marie-laure.sobrier@inserm.fr (M.-L.S.); 2 Assistance Publique-Hôpitaux de Paris (AP-HP), Sorbonne University, F-75012 Paris, France

**Keywords:** *IGF2*, growth, Silver–Russell syndrome, Beckwith–Wiedemann syndrome, parental imprinting

## Abstract

In the 30 years since the first report of parental imprinting in insulin-like growth factor 2 (*Igf2*) knockout mouse models, we have learnt much about the structure of this protein, its role and regulation. Indeed, many animal and human studies involving innovative techniques have shed light on the complex regulation of *IGF2* expression. The physiological roles of IGF-II have also been documented, revealing pleiotropic tissue-specific and developmental-stage-dependent action. Furthermore, in recent years, animal studies have highlighted important interspecies differences in IGF-II function, gene expression and regulation. The identification of human disorders due to impaired *IGF2* gene expression has also helped to elucidate the major role of IGF-II in growth and in tumor proliferation. The Silver–Russell and Beckwith–Wiedemann syndromes are the most representative imprinted disorders, as they constitute both phenotypic and molecular mirrors of *IGF2*-linked abnormalities. The characterization of patients with either epigenetic or genetic defects altering *IGF2* expression has confirmed the central role of IGF-II in human growth regulation, particularly before birth, and its effects on broader body functions, such as metabolism or tumor susceptibility. Given the long-term health impact of these rare disorders, it is important to understand the consequences of *IGF2* defects in these patients.

## 1. Introduction

Insulin-like growth factor two (IGF-II) is a key protein regulating growth, particularly during normal fetal development, but it is also often dysregulated during tumorigenesis [1,2,3]. In this review, we focus on the role of IGF-II in physiological functions and its regulation by *cis* or *trans* genetic factors. We do not consider here the role of IGF-II in the context of tumors, or its regulation by environmental factors.

**Figure 1:**
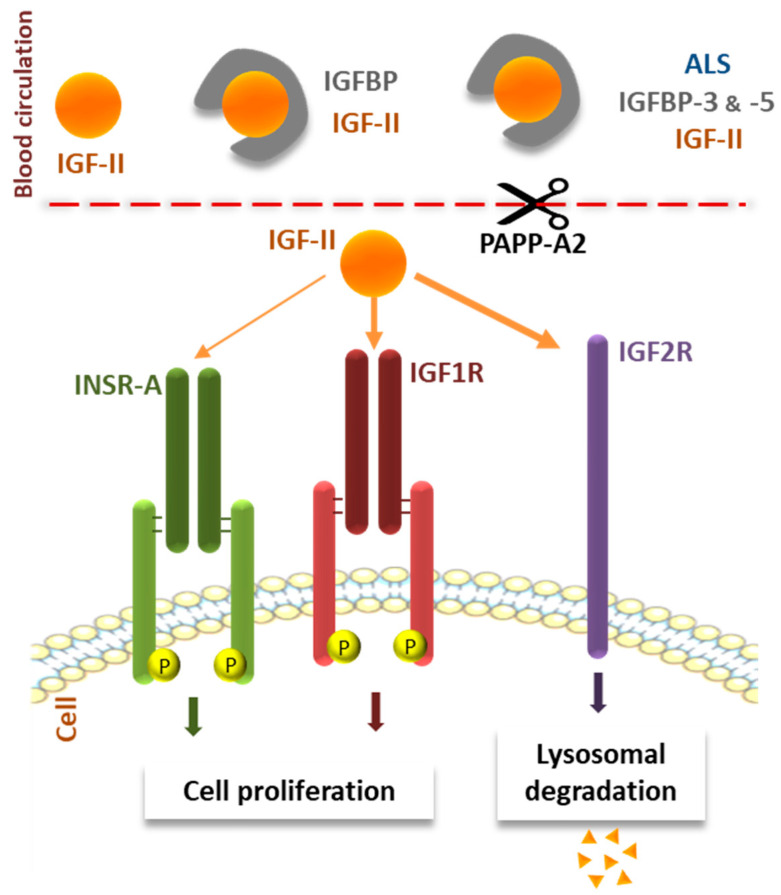
Schematic representation of the modes of action of IGF-II. In the bloodstream, IGF-II is mostly bound in a ternary complex with the acid-labile subunit (ALS) and IGF-binding proteins (IGFBP)-3 and -5. Once released from this complex by PAPP-A2 proteolysis, IGF-II can bind either the type A insulin receptor (INSR-A) or IGF receptor type 1 or 2 (IGF-1R and IGF-2R), inducing cell proliferation or IGF-II clearance.

IGF-II belongs to a larger system including several different regulatory factors and generally referred to as the “IGF system” that we will briefly present. IGF-I and IGF-II are the main ligands of the type 1 IGF receptor (IGF-1R). This tyrosine kinase receptor is composed of an extracellular domain consisting of an alpha chain, a transmembrane domain, and an intracellular domain consisting of a beta chain carrying the tyrosine kinase sites. The ligand binds dimerized IGF-1R and transmit the resulting signal (Figure 1) [4]. 

The binding of IGF-I or IGF-II to IGF-1R leads to activation of the downstream MAP kinase and PI3 kinase signaling pathways [5,6]. IGF-1R has a higher affinity for IGF-I than for IGF-II (K_d_ = 1.5 nM and 3.0 nM, respectively) [7,8]. Thanks to its high degree of similarity to IGF-1R, the insulin receptor (INSR) can also bind IGFs, but with negligible affinity for IGF-I, and with only type A INSR having a five-fold lower affinity for IGF-II than for insulin [9,10]. Another receptor, the type 2 IGF receptor (IGF-2R), which is a mannose-6-phosphate cation-dependent receptor, also specifically binds IGF-II [11,12,13]. Its role has been demonstrated in IGF-II clearance through lysosomal degradation and more recent studies in rodents stated for its role in memory enhancement processes [14,15,16,17,18]. The plasma half-lives of both IGF-I and IGF-II are extended by binding proteins (IGFBP). Six different IGFBPs have been identified, each with relative different affinities between IGFs but binding IGFs with a higher affinity than IGF-1R [19,20,21,22,23]. IGFBP-3 and -5 can also form a larger complex with the acid-labile subunit (ALS), which can bind IGFs, increasing the stability of these factors in the blood [24]. The bioavailability of IGFs is dependent from the homeostasis between their bound and free forms which is regulated through IGFBP proteolysis [23,25,26,27,28]. As an illustrating example, PAPP-A2 (Pregnancy-associated Plasma Protein-A2) is a metalloproteinase which specifically cleaves IGF from IGFBP-3 and -5 to allow IGF activity [29,30]. Recently, pathogenic variation in *PAPPA2* has been reported in growth-retarded children resulting from decreased IGF bioactivity [31].

IGF-II is secreted, mostly via the placenta, during pregnancy [32,33]. After birth, the IGF-II circulating in the bloodstream for the greater part results from secretion from hepatocytes, but unlike that of IGF-I, this secretion is not dependent on growth hormone (GH) secretion [34,35].

**Figure 2:**
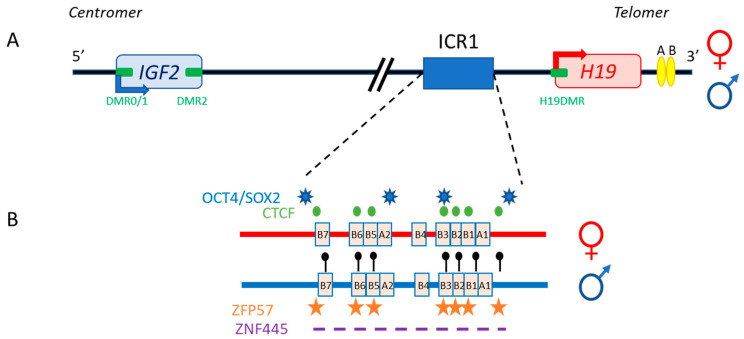
The human *IGF2/H19* 11p15.5 locus. (**A**) The *IGF2* and *H19* genes are separated by about 80 kb. *IGF2* is paternally expressed (blue arrow), whereas *H19* is maternally expressed (red arrow). The four DMR (green boxes) and enhancers (yellow ellipses) are represented. (**B**) *H19/IGF2*:IG-DMR (ICR1) in detail: OCT4/SOX2 (blue stars) and CTCF (green circles) binding sites on the maternal allele (red), and methylation sites (black lollipops), ZFP57 binding sites (orange stars) and the undefined ZNF445 binding site consensus sequence (purple dash) on the paternal allele (blue) are shown.

IGF-II synthesis results from the expression of *IGF2*, an imprinted gene located in the chromosome 11p15.5 region. Imprinted genes are characterized by their monoallelic expression, which is dependent on the parental origin of the allele. This pattern of expression is controlled by epigenetic marks on differentially methylated regions (DMR) known as imprinting control regions (ICR) [36,37]. In the 11p15.5 region, *H19/IGF2*: InterGenic-DMR (IG-DMR or ICR1) is methylated on the paternal allele, driving the expression of the paternal *IGF2* allele, whereas the absence of methylation on the maternal allele leads to the expression of *H19*, a non-coding transcript (Figure 2).

## 2. Structural and Regulation Aspects

### 2.1. Main Characteristics, Linear Organization

In 2019, Baral et al. unraveled the complexity of the genomic and transcriptional organization of the *IGF2/Igf2* locus, by using human or mouse DNA segments as queries in genome analyses, and RNA sequencing libraries (complete review in [38]). *IGF2* (ENSG00000167244) is composed of 10 exons and five promoters, whereas its mouse counterpart is located on chromosome 7, and is composed of eight exons and four promoters.

**Figure 3:**
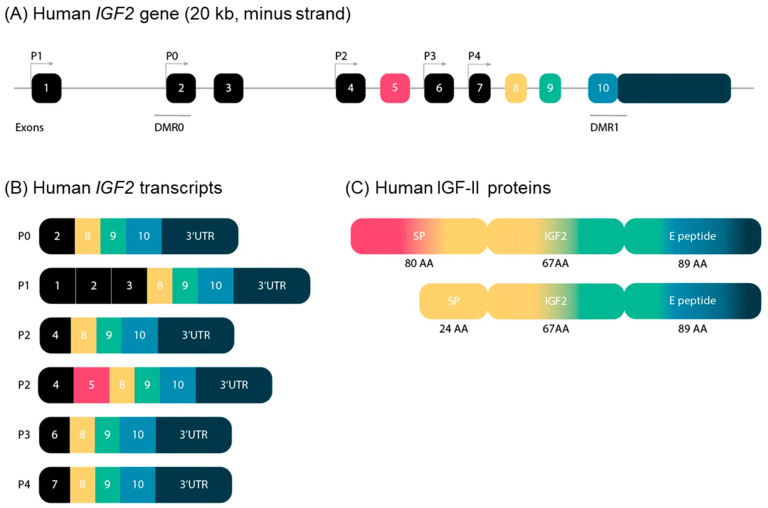
(**A**) Schematic representation of the structure of the *IGF2* gene in humans. The *IGF2* gene consists of 10 exons and is driven by five different promotors. The exons of the *IGF2* gene are boxed. The black boxes indicate non-coding exons. The colored boxes indicate the coding exons. The turned arrows show the promotors (P) and indicate the transcription start sites. The blue lines indicate the differentially methylated regions (DMR) in the *IGF2* gene. (**B**) Transcripts of the human *IGF2* gene. *IGF2* has six alternative transcripts, depending on promotors and splice sites used. (**C**) Human IGF-II proteins. IGF-II has two precursor proteins. Only exons 5, 8, 9 and 10 encode IGF-II proteins. Exon 5 is not included in the composition of the second precursor protein, which therefore has a smaller signal peptide. SP: signal peptide; AA: amino acids.

The five human *IGF2* promoters control the expression of different non-coding exons, but all transcripts include exons 8–10, which encode the IGF-II protein precursor and the 3′ untranslated RNA. Human promoters 1 and 2 (P1 and P2) are species specific, and P2 regulates two classes of *IGF2* transcripts differing due to alternative splicing of exon 5 (Figure 3).

The human-secreted IGF-II is composed of 67 amino acids organized into four domains, the B, C, A, and D domains (listed in order from the N- to the C-terminus) [39]. Two types of protein precursors with different presumptive N-terminal signal peptides (consisting of 24 or 80 amino acids) give rise to mature human IGF-II, depending on the inclusion or exclusion of exon 5 in the *IGF2* mRNA. The E peptide of the IGF-II precursor (also named “big” IGF-II), encoded by the 3-’end mRNA, is 89 amino acids long (Figure 3). It has been involved in paraneoplastic pancreatic independent hypoglycemia [40,41].

The 11p15.5 locus also includes *H19*, which is separated from *IGF2* by about 80 kb (Figure 2). In both humans and mice, *IGF2* and *H19* are imprinted in a reciprocal manner. Moore et al. detected three *Igf2* antisense transcripts relative to P0 transcription, with no open reading frames, in mice [42]. The exact role of these transcripts is unclear, and it is unknown whether their co-expression with the sense transcript (which has yet to be demonstrated), within the same cell, would influence mRNA stability.

Different protein-binding sites are involved in regulating the expression of these two genes. The first evidence of differences in protein-binding sites and methylation status between the two alleles came from DNA hypersensitive site studies on the mouse *Igf2/H19* locus [43]. These studies revealed clear differences in nuclease sensitivity between the parental chromosomes, with the presence of mutually exclusive hypersensitive sites (on the maternal chromosome) and DNA methylation sites (on the paternal chromosome).

**Figure 4:**
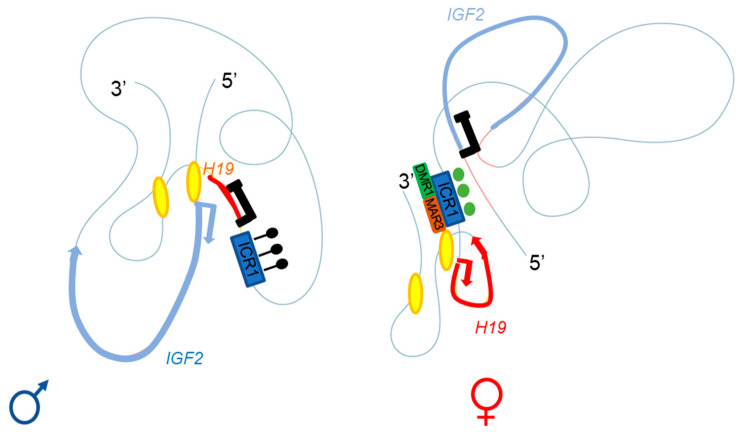
Adapted from Rovina et al. [60]. Three-dimensional representation of the *IGF2-H19* locus on the paternal (**left**) and maternal (**right**) chromosomes. The ICR1 of the paternal allele is methylated (black lollipops), with enhancers A and B (yellow ellipses) close to the *IGF2* promoter; this conformation allows *IGF2* expression and *H19* repression. The CTCF of the maternal allele can bind the unmethylated ICR1 (green circles), regulating the interaction with DMR1 (green box) and the matrix attachment region (MAR)3 (brown box); the A and B (yellow ellipses) enhancers are close to the *H19* promoter. This conformation allows *H19* expression and *IGF2* repression.

The specific parent-of-origin pattern of expression of *H19* and *IGF2* at 11p15.5 is controlled by the allele-specific methylation status of *H19/IGF2*:IG-DMR. This locus is composed of seven CTCF-binding sites (CBS1-7) located in the A and B blocks of repeated domains (Figure 2). CTCF is a highly conserved zinc-finger DNA-binding protein with multiple roles in gene regulation [44]. The region orthologous to ICR1 in mouse contains only four CBS at the *Igf2* locus. Several studies in humans have reported methylation at all CBS in *H19/IGF2*:IG-DMR on the paternal allele and established a correlation between the methylation of these CBS and *IGF2* expression from the paternal allele [45]. Our team has also demonstrated homogeneous methylation levels for all CBS in humans [46]. In mice, CTCF binding to the unmethylated maternal ICR is essential for imprint maintenance in somatic cells, providing protection against aberrant de novo methylation at DMR throughout the locus. Furthermore, CTCF acts as an insulator, and CTCF binding creates a small loop of silent chromatin (CTCF binding to the maternal ICR regulates its interaction with matrix attachment region (MAR)3 and DMR1 at Igf2) (Figure 4), preventing enhancers from gaining access to the *Igf2* promoter [47]. *H19/IGF2*:IG-DMR methylation on the paternal allele abolishes CTCF binding and ICR-mediated insulation, resulting in functional communication between promoters and enhancers, and an activation of *Igf2* expression (see below).

In addition to CBS, the human IGF2/H19 domain contains four binding sites for the pluripotency factors OCT4 and SOX2 [48]. There is evidence to suggest that the function of CTCF is modulated by the binding of OCT4/SOX2 to neighboring areas of DNA [49]. These factors play a role in maintaining or establishing the unmethylated status of the maternal allele. This hypothesis is strongly supported by in vitro experiments and by studies in transgenic mouse models showing that the OCT4/SOX2 binding sites in the maternal *H19/IGF2*:IG-DMR are essential for the full protection of DNA methylation during the establishment or maintenance phases [50,51]. Indeed, in humans, mutations of these OCT4/SOX2 binding sites in the maternal allele lead to hypermethylation of the CBS, followed by an increase in *IGF2* expression leading to Beckwith–Wiedemann syndrome [52]. Two other factors, ZFP57 and ZNF445, protect ICR from DNA demethylation after fertilization. ZNF445 seems to be sufficient on its own in humans, whereas ZFP57 and ZNF445 cooperate in rodents (Figure 2) [53,54]. The *IGF2/H19* domain also contains several other DMR: three within the *IGF2* gene (DMR0, DMR1 and DMR2) and an additional DMR located in the *H19* promoter (*H19*DMR), all of which are secondary DMR (somatic DMR that acquire their parent-specific DNA methylation mark in somatic diploid cells) methylated on the paternal allele [55] (Figure 2).

### 2.2. Three-Dimensional Organization

The regulation of *IGF2* expression must be considered in three dimensions, with CTCF playing a major role in this aspect. There is experimental evidence to suggest that CTCF confers allele-specific effects on transcription via long-range chromatin interactions. The generation of these data was made possible by the emergence of 3C technology [56]. These interactions are dependent on the parental origin of the chromatin.

Series of deletions at the *H19/Igf2* locus have made it possible to demonstrate the presence of several enhancers, two of which are predominantly endodermal and located 10 kb from the start site of the *H19* transcript. These two enhancers target the *H19* and *Igf2* promoters, allowing expression of the corresponding genes (see below and Figure 2 and Figure 4) [57]. For the maternal allele, for which *Igf2* expression is silent, 3C data are generally consistent with a model in which the CTCF-bound ICR contacts both the upstream DMR1 and a downstream matrix attachment region (MAR). Genetic studies have confirmed that CTCF binding to the ICR is required for both the formation of ICR-DMR1-MAR contacts and the prevention of maternal-specific enhancer-*Igf2* promoter interactions. By contrast, on the paternal allele, which displays active *Igf2* expression, all DMR sequences are methylated, preventing CTCF binding, and most of this region appears to be accessible, allowing more fluid contact with the enhancers [47,58].

Recent efforts to elucidate chromatin organization at the *Igf2*/*H19* mouse locus, based on a combination of studies of allelic CTCF binding with both high-resolution and single-cell 3D chromatin organization assays, defined topologically associated domains (TAD) [59]. These studies determined the dynamic structure of the imprinted *Igf2-H19* domain, and showed that CTCF binding occurred at multiple sites in both alleles, exclusively in ICR1 for the maternal. Furthermore, combinations of allelic 4C-seq and DNA-FISH revealed that CTCF binding to the paternal chromosome alone was correlated with a first level of sub-TAD structure. Additional CTCF binding to the differentially methylated region on the maternal chromosome adds a further layer of sub-TAD organization. This allele-specific sub-TAD organization may, thus, provide an instructive or permissive context for the correct activation of imprinted genes during development.

In humans, a specific parent-of-origin pattern of expression through TAD generation according to CTCF binding has been described, leading to *IGF2* expression or silencing [60]. As in mice, the most important proteins for TAD architecture are CTCF (which bind to the ICR) (Figure 4). Moreover, crosstalk between *IGF2/H19* and the *CDKN1C/KCNQ1OT1* domain (another imprinted domain located in the same chromosome region) has been detected on the basis of a higher order of chromatin folding, suggesting the involvement of a mechanism for coordinating the expression of genes with the same expression status: *IGF2* and *KCNQ1OT1* on the paternal allele and *H19* and *CDKN1C* on the maternal allele [60].

### 2.3. Trans-Regulation Mechanisms

In addition to being regulated by *H19/IGF2*:IG-DMR methylation and the three-dimensional organization of chromatin, *IGF2* can be directly regulated through the activation of its promoters by several transcription factors, including those of the oncogenic HMGA2-PLAG1 pathway [61]. *PLAG1* (pleiomorphic adenoma gene 1) overexpression was first observed in pleiomorphic adenomas of the salivary glands, identifying *PLAG1* as an oncogene [62]. PLAG1 is a nuclear factor with seven zinc-finger domains that can bind *IGF2* promoter P3, upregulating its transcriptional activity [62]. This finding has been confirmed in various other tumors, including hepatoblastomas, lipoblastomas and leukemia (review in [63]). Interestingly, *Plag1* inactivation in mouse models results in pre- and postnatal growth retardation, despite an absence of change in *Igf2* expression in embryos and pups [64].

HMGA2 (high mobility group AT-hook 2), initially named HMGI-C, is a member of the high-mobility group of proteins. Its expression is usually barely detectable in normal adult cells, but increases in cells transformed with viral oncogenes and in malignant tissues [65]. Mouse models of *Hmga2* inactivation have a pygmy phenotype, with pre- and postnatal growth restriction and craniofacial abnormalities (a shortened head) [66]. The expression levels of *HMGA2* and *PLAG1* are highly correlated in thyroid tumors, and *HMGA2* overexpression in cellular models is associated with an increase in *PLAG1* expression [67].

The role of this oncogenic pathway in the control of *IGF2* expression was highlighted in 2017, with the identification of additional mutations of *HMGA2* and the first mutations of *PLAG1* in patients referred for Silver–Russell syndrome (in addition to original mutations of *IGF2*). One of these *PLAG1* mutations led to a downregulation of *IGF2* expression in fibroblasts through a specific change in P3 promoter activity. Finally, the overexpression of *HMGA2* and *PLAG1* or their silencing in transfection assays result in a gain in expression or the downregulation of *IGF2* expression, respectively [61].

*DIS3L2* (*DIS3-like 3′-5′ exoribonuclease 2)* encodes a protein involved in the processing of mRNA and small non-coding RNAs. Homozygous loss-of-function variants of *DIS3L2* lead to a rare condition called Perlman syndrome. This syndrome is characterized by excessive fetal growth and an increase in the risk of Wilms’ tumor [68]. In a mouse model, *Dis3l2* invalidation was associated with an overexpression of *Igf2* in nephron progenitor cells that was not associated with a loss of imprinting, as *Igf2* still displayed monoallelic expression. The mechanism of *Igf2* overexpression in this model remains to be determined [69].

Network of imprinted genes: In recent years, several studies in humans or animal models have shown that abnormalities at a given imprinted locus can impact at the expression of genes not only at the locus concerned, but also at other imprinted or non-imprinted loci [70,71,72,73]. This finding raised the possibility of an imprinted gene network, within which, imprinted genes are co-regulated. This pattern of regulation may partly account for the clinical overlap between imprinting disorders due to (epi)genetic defects at different imprinted loci [74]. For example, a strong clinical overlap between Silver–Russell syndrome (SRS, OMIM #180860) and Temple syndrome (TS14, OMIM #616222) has been described, despite the existence of several syndrome-specific traits, including pre- and postnatal growth restriction, relative macrocephaly, feeding difficulties and a protruding forehead [75]. *IGF2* downregulation is thought to be the molecular mechanism underlying the SRS phenotype, with about 40% of SRS patients presenting hypomethylation at the *H19/IGF2*:IG-DMR [76]. TS14 is mostly due to abnormalities of the imprinted 14q32.2 locus. This locus contains non-coding RNA sequences that are expressed from the maternal allele only (including the two long non-coding RNA, *MEG3* and *MEG8*). In cases of maternal uniparental disomy of chromosome 14 or hypomethylation at the *MEG3/DLK1*:IG-DMR, *MEG3* and *MEG8* are expressed from both the paternal and maternal alleles, leading to an increase in the level of expression of these two genes. Abnormally low levels of *IGF2* expression have been reported in the fibroblasts of TS14 patients, despite normal *H19/IGF2*:IG-DMR methylation. Furthermore, in control fibroblasts, the overexpression of *MEG3* and *MEG8* leads to a downregulation of *IGF2*. Conversely, the silencing of *MEG3* and/or *MEG8* in control fibroblasts leads to an upregulation of *IGF2* expression. Thus, *MEG3* and *MEG8*, which are expressed from the maternal 14q32.2 locus, regulates *IGF2* expression at 11p15.5, providing support for the hypothesis of an imprinted gene network [77].

## 3. Physiological Roles

### 3.1. IGF-II: A Key Factor in Development

**Figure 5:**
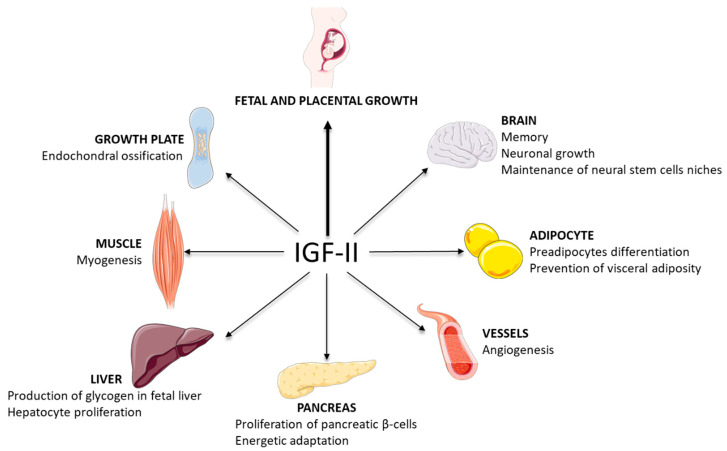
Diagram summarizing the physiological roles of IGF-II described in humans and mouse models.

IGF-II is a growth factor with a structural and regulatory complexity associated with pleiotropic tissue-specific and developmental-stage-dependent action (Figure 5).

Paternally expressed imprinted genes are usually associated with a pro-proliferative role during development. This is particularly true for the *IGF2* gene, which encodes a key factor for feto-placental growth [78]. Indeed, IGF-II is highly mitogenic, and together with IGF-I, it promotes the proliferation of various types of cells during the fetal period, thereby playing a major role in organ growth and development.

In mice, the *Igf2* gene plays a crucial role during the embryonic period. The P0 promoter operates specifically in the placenta, leading to extremely high levels of *Igf2* expression in placental tissues during gestation. The complete inactivation of *Igf2* (*Igf2* null^+mat/−pat^), and specific inactivation of the placental transcript *Igf2*-P0 (*Igf2* P0^+mat/-pat^) have been studied experimentally in mice. *Igf2*-null mice display intrauterine growth restriction and placental hypoplasia. Furthermore, the specific inactivation of *Igf2*-P0 leads to intrauterine growth restriction through placental restriction [36,79]. These mouse models also present changes to feto-maternal exchanges, including, in particular, the supply of maternal nutrients to the fetus [1,80]. These alterations can be explained by the role of the Igf2/Igf2R axis in placental vascularization and the adaptation of the placenta to fetal needs [81].

However, there are important differences between humans and mice, particularly in the placenta. For example, the human placenta is monochorial, with interanvil spaces, whereas the mouse placenta is trichorial and has a labyrinth region. Moreover, the human placenta is associated with some extremely specific molecular patterns, such as the expression of certain genes (*miR-194*, *C19orf33*, *SIGLEC6*, *estrogens*, *glycodelin A*, *chorionic gonadotropins*, etc.) [82,83]. Furthermore, in humans, *IGF2*-P0 is not specific to the placenta and is expressed in other tissues, including skeletal muscle. There is no clear evidence to suggest that the mouse model is strictly comparable to humans, so it remains unclear whether the same placental dysfunctions occur in mice and humans [55,84]. Data obtained from human placenta explants and the human BeWo cell (a choriocarcinoma cell line) model suggest that abnormalities in the functioning of the IGF system, including IGF2R impairment, in particular, would lead to an imbalance between proliferation and apoptosis in trophoblasts [85]. In addition, patients with SRS (i.e., with low levels of *IGF2* expression, see below) display hypoplasia of the placenta and chorionic villi. Moreover, this hypoplasia is commonly associated with oligohydramnios, consistent with placental dysfunction in this syndrome [84]. By contrast, a study on patients with Beckwith–Wiedemann syndrome (BWS, #130650, see below) with various molecular etiologies showed that most individuals with BWS, which is caused by *IGF2* overexpression, displayed placentomegaly [86,87]. These studies clearly show that deregulations of the *IGF2* and IGF system can cause changes to placental structure and function in humans [85]. *IGF2* is also highly expressed in fetal tissues, under the control of various promoters, depending on the tissue concerned, and is involved in the maturation and development of mesoderm-derived tissues, in particular [88,89,90,91].

In mice, *Igf2* expression decreases rapidly in all tissues after birth, potentially accounting for the intrauterine growth phenotype but minimal effects on postnatal growth in *Igf2*-null mice [36,92]. However, *Igf2* expression is maintained in the brain, particularly in the hippocampus, where it plays a role in memory processes, learning and brain plasticity [18,93,94]. This postnatal expression of *Igf2* is also involved in homeostasis of stem cells niches in brain and intestine [95].

In humans, serum IGF-II concentration remains high (400–1000 ng/mL) during the postnatal period, despite the significant decrease in *IGF2* expression observed in tissues. The exact physiological role of this circulating IGF-II and the absence of interference with the GH-IGF-I regulation, despite the fact that IGF-I and IGF-II have fairly similar affinities for IGF1R remains to be elucidated.

The IGF-II in serum is produced by the liver under the control of the P1 promoter and released into the bloodstream. The difference in postnatal *IGF2* expression between humans and mice is thought to be due to the presence of the *IGF2* P1 promoter in humans, and its absence in mice [96]. During the postnatal period, the P3 and P4 promoters are responsible for *IGF2* expression in most tissues [34,89,96]. Interestingly, the P1 promoter is not imprinted (*IGF2*-P1 expression is, therefore, biallelic) and has been reported to be liver-specific, although doubts have been raised about this specificity following the demonstration that cells in other tissues, such as chondrocytes, express the *IGF2*-P1 transcript [34,96,97,98]. *IGF2* displays monoallelic expression in the fetal liver, and is dependent mainly on the P3 and P4 imprinted promoters.

### 3.2. Some Roles of IGF-II in Tissues

The other roles of IGF-II in tissues, apart from those cell proliferation and organ growth, remain unclear. Many studies have sought to elucidate the role of IGF-II in the development, maintenance and function of various tissues. Loss- or gain-of-function models (mice or cellular models) have been developed to shed light on the mechanism of action of IGF-II.

Several studies in mice have shown that Igf-II is involved in endochondral ossification within the growth plate, which governs bone growth. Autocrine Igf-II in the growth plate activates the PI3K/Akt and TGF-β signaling pathways, leading to the expression of proliferative factors that stimulate chondrocyte proliferation, pro-osteogenic factors, such as BMP-9 and alkaline phosphatase, which mediate ossification, and constituents of cartilage, such as proteoglycans. Igf-II has also been implicated in partial regulation of the development and organization of the growth plate, through the regulation of glucose metabolism [99,100,101].

Other studies have revealed the role of IGF-II in angiogenesis. Igf-II promotes the mesodermal, and then endothelial differentiation of mouse embryonic stem cells. In HUVEC cells, IGF-II activates sprouting, leading to the activation of endothelial cells and vasodilation. IGF-II and IGF-1R are essential for the maintenance of tip cells, a particular type of endothelial cell responsible for guiding de novo angiogenesis. The binding of IGF-II to IGF1-R activates the PI3K/Akt signaling pathway, switching on the cell migration programs necessary for angiogenesis. IGF-binding proteins, such as IGFBP-3 and IGFBP-4, modulate the bioavailability of IGF-II, which regulates the effect of IGF-II on sprouting angiogenesis. IGF-II is also involved in angiogenesis through its role in maintaining hypoxia-induced factor α (*HIF-α*) levels, leading to expression of the vascular endothelial growth factor (*VEGF*) gene [102,103,104].

In human adipose tissue, IGF-II induces the differentiation of subcutaneous preadipocytes and inhibits the differentiation of visceral preadipocytes. IGF-II also downregulates the insulin receptor IR-A and expression of the glucose transporter GLUT4 in visceral adipocytes, thereby preventing the development of adiposity in visceral compartments [105].

In mouse fetal liver, Igf-II regulates glycogen production. Igf-II binds type A INSR, which activates the PI3K/Akt pathway, leading to the phosphorylation of glycogen synthase, which catalyzes the production of glycogen in fetal liver [106]. Igf-II is also involved in hepatocyte proliferation in mice [107].

Some studies on mouse models have postulated a role for Igf-II in the regulation of pancreatic size and function. Indeed, Igf-II synthesized in the pancreatic mesenchyme exerts a paracrine effect on the proliferation of pancreatic β-cells and, thus, on the size and function of the exocrine pancreas during the pre- and postnatal periods. By contrast, the autocrine Igf-II produced by pancreatic β-cells plays a role in adaptation to energy demands in pregnant mice [108,109,110].

One of the best-known and most-studied roles of IGF-II is that in myogenesis. IGF-II plays a direct role in the differentiation of mesoderm into myoblasts by upregulating the expression of MyoD, a key factor determining musculoskeletal fate. It also acts on striated skeletal muscle homeostasis and, thus, on the maturation, maintenance and healing of such muscles. In skeletal muscle, *IGF2* transcription depends on the P3 promoter and the mTOR protein. IGF-II acts by binding to the IGF-1R, thereby triggering the PI3K/Akt signaling cascade required for the differentiation of mesenchymal stem cells into musculoskeletal cells [111,112,113]. Several microRNAs, such as miR-223 and miR-125b, play a role in skeletal muscle homeostasis by modifying *IGF2* expression and, thus, its action on myoblast proliferation and differentiation [114,115].

The role of IGF-II in the brain remains to be clearly defined in humans, but studies on rodent models have shown that Igf-II makes a major contribution to the correct functioning of the brain. Igf-II is the most abundant Igf in the adult rodent central nervous system, with particularly high levels in the hippocampus. Moreover, Igf-2R is expressed in almost all regions of the brain, including the hippocampus, olfactory bulb, dentate gyrus, choroid plexus, and the cerebral vascular system. Thus, by regulating Igf-II bioavailability, Igf-2R controls not only neuronal growth and differentiation, but also the mechanisms of neuronal regeneration. Moreover, Igf-I and Igf-II control neuronal survival by inhibiting apoptosis [116]. Studies in mouse models have also revealed a role for Igf-II in the maintenance of adult neural stem-cell niches. Thus, *Igf2* deletion results in the differentiation of neural stem cells into neurons, leading to hyposmia, due to an increase in the number of neurons in the olfactory bulb, together with cognitive deficits and increased anxiety [95].

Studies in rats have shown that Igf-II is involved in hippocampus-dependent learning mechanisms. Indeed, Igf-II plays an important role in the consolidation of memory, the retention of information and the prevention of forgetfulness phenomena. These effects are mediated by the *Igf2* receptor and lead an increase in the expression of the AMPA receptor subunit GluA1 (ionotropic receptor for glutamate heavily involved in synaptic plasticity) at the synapses, and to activation of the glycogen synthase kinase 3-β enzyme [17,18]. These phenomena participate in the long-term potentiation (LTP) responsible for memory consolidation.

Conversely, several studies in mice have suggested that the deregulation of *Igf2* expression may contribute to certain mental illnesses, leading to the hypothesis of a link between decreases in hippocampal Igf-II levels and increases in anxious and depressive behaviors [117,118]. In humans (post-mortem analysis), *IGF2* downregulation in the prefrontal cortex is associated with schizophrenic disorders [119,120,121]. Studies on mice and cell models derived from mice have shown that the misregulation of *Igf2* is associated with autistic behavior and neurodegenerative diseases, such as Huntington’s disease and Charcot’s disease. In these two neurodegenerative diseases, *Igf2* stimulation has a positive effect by preventing the degeneration of motor neurons and promoting their regeneration [122,123,124]. In autistic disorders, *Igf2* stimulation leads to a reversal of the clinical signs of autism (restoration of social behavior, abolition of repetitive behaviors, etc.) [125]. Few cognitive studies have been performed in SRS patients with low levels of *IGF2* expression, and one study showed that SRS patients did not actually present cognitive deficits relative to a control population, but that they had a smaller frontal and parietal lobe volume in the brain [126].

## 4. Pathological Aspects

### 4.1. Silver–Russell Syndrome (SRS)

SRS is a well-recognized imprinting disorder including prenatal and postnatal growth retardation. Clinical diagnosis is currently based on a combination of the characteristic features evaluated with a clinical scoring system (Netchine–Harbison Clinical Scoring System, NHCSS) [127]. Relative macrocephaly at birth is a key criterion for diagnosis and exposes the patient to a high risk of hypoglycemia, which should be carefully monitored. The first international consensus conference on SRS was held in 2015 [76]. A molecular abnormality can be identified in about 60% of patients with a positive clinical diagnosis of SRS (NHCSS > 3). The main molecular causes are low levels of *IGF2* expression, due to a loss of methylation of the distal imprinting control region (*H19/IGF2*:IG-DMR) on 11p15.5 (50%), other rare 11p15.5-related molecular defects, such as *IGF2* point mutations affecting the paternal allele, mutations or deletions of *HMGA2* and *PLAG1*, or gain-of-function mutations of *CDKN1C* [61,128,129,130,131,132]. However, after birth, serum IGF-II levels are within the normal range. Indeed, the IGF-II in the serum, which is principally of hepatic origin, results from biallelic *IGF2* expression regulated by the P1 promoter. However, as pointed out above, *IGF2* remains imprinted and its expression in other tissues is monoallelic and regulated by the P3 and P4 promoters [130].

SRS patients require multidisciplinary care, as they have many different health issues, including growth failure, severe feeding difficulties in early childhood, gastrointestinal problems, hypoglycemia, puberty and reproductive disturbances, motor and speech delay, sleep apnea and psychosocial challenges [76]. They have also been reported to experience metabolic disturbances in early adulthood, illustrating Barker’s developmental origin of health and diseases theory, according to which, fetal growth retardation triggers long-term health issues [133,134,135], and suggesting that low levels of *IGF2* expression during fetal development may have long-term consequences for key physiological processes.

### 4.2. Temple Syndrome (TS14)

The phenotypes of TS14 and SRS overlap [75,136,137]. TS14 is characterized by pre- and postnatal growth failure, albeit not as severe as in SRS. Fetal growth restriction may be present in up to 75% of cases, a frequency similar to that in SRS. About 50% of TS14 patients have relative macrocephaly at birth. Severe neonatal-onset hypotonia is a prominent feature. Early obesity and precocious puberty onset are typical (86%), often requiring treatment with gonadotropin-releasing hormone analogs [75]. The molecular abnormalities underlying TS14 include hypomethylation of the *MEG3/DLK1*:IG-DMR in the human 14q32.2 imprinted region. As pointed out above, the downregulation of *IGF2* expression in the fibroblasts of TS14 patients may account for the clinical overlap between TS14 and SRS [77].

### 4.3. Wilms’ Tumors and Beckwith–Wiedemann Syndrome

It has been known for decades that *IGF2* is overexpressed in Wilms’ tumors relative to normal postnatal kidney [138]. A loss of heterozygosity (i.e., loss of the maternal allele) or loss of imprinting at the *H19/IGF2*:IG-DMR (i.e., biallelic expression of *IGF2*) in Wilms’ tumors was subsequently demonstrated by several teams [139,140]. Finally, in 1995, Taniguchi et al. showed that the loss of *IGF2* imprinting in Wilms’ tumors was associated with hypermethylation at the *H19/IGF2*:IG-DMR, as reported in patients with BWS [141].

Beckwith–Wiedemann syndrome is an overgrowth syndrome. The patients often present with macroglossia, abdominal wall defects, hemihyperplasia, enlarged abdominal organs, and a high risk of embryonal tumors (especially Wilms’ tumors) during early childhood. BWS is mostly due to genetic or epigenetic defects in the 11p15.5 region. The first consensus statement on BWS was released in 2016 [142]. Various molecular defects were identified, including mosaic segmental paternal uniparental isodisomy of 11p15.5 (commonly referred to as segmental upd(11)pat), which can be detected in 20% of patients, and gain of methylation (GOM) at the maternal *H19/IGF2*:IG-DMR allele, which is present in 5–10% of cases. Both lead to *IGF2* overexpression during fetal life. In addition to molecular abnormalities of the *IGF2* locus, abnormalities of the *CDKN1C* locus (i.e., hypomethylation of the *KCNQ1OT1*:TSS-DMR or maternal loss-of-function mutations of the *CDKN1C* gene) account for about 75% of the molecular defects. Interestingly, tumor risk is highly correlated with the involvement of the *H19/IGF2* locus, as patients with *H19/IGF2*:IG-DMR GOM or with upd(11)pat have tumor risks of 28% and 16%, respectively, whereas patients with *KCNQ1OT1*:TSS-DMR LOM have a much lower prevalence of tumors, at 2.6% [143]. *IGF2* overexpression, as observed in BWS, is thus responsible for macrosomia, organomegaly and an increase in the risk of embryonal tumors.

## 5. Conclusions

*IGF2* belongs to an imprinted gene network. Its expression is regulated by several upstream factors, and it regulates numerous downstream effectors. The application of new high-throughput technologies (i.e., next-generation sequencing, evaluations of DNA methylation and RNA sequencing) to imprinted disorders in mice or induced pluripotent stem cells and promising dental pluripotent stem cells models should make it possible to decipher more precisely the upstream and downstream actors involved in multiple tissue-specific functions of IGF-II [144,145]. The potential breakthroughs associated with such modeling of *IGF2*-linked diseases open up new challenges and expand this field of research still further.

## Data Availability

Not applicable.

## Nomenclature

IGF-II (IGF-I, IGF-1R, etc.) is used for the protein in humans, and Igf-II (Igf-I, Igf1r, etc.) is used in other species. *IGF2 (IGF1*, *IGF1R*, etc.) is used for the gene in humans, and *Igf2* (*Igf1*, *Igf1r*, etc.) is used in other species.
